# How to measure omega-3 fatty acid lysolipid flipping: The ultimate guide

**DOI:** 10.1073/pnas.2302321120

**Published:** 2023-03-27

**Authors:** Camilo Perez

**Affiliations:** ^a^Biozentrum, University of Basel, Basel 4056, Switzerland

Major facilitator superfamily (MFS) transporters are responsible for the translocation of a vast amount of highly diverse substrates across biological membranes. During the last two decades, structures of about 40 MFS transporters, elucidated using X-ray crystallography or cryogenic electron microscopy methods, gave insight into how MFS transporters cycle through distinct conformations achieving vectorial transport of substrates (1). A large majority of the structurally characterized MFS transporters catalyze the transport of polar hydrophilic substrates, such as monosaccharides and disaccharides, amino acids, and nucleosides. However, it was until very recently that structures of MFS transporters involved in the transport of lipids have been determined (2–5), bringing to light mechanistic models that could answer questions such as, how is a lipid substrate recognized by an MFS transporter? What is the mechanistic role of ion coupling when the substrate is a lipid? How does gating work when the substrate is a molecule that moves laterally in the membrane bilayer?. However, advancing our knowledge on how these proteins work not only relies on elucidation of structures but also on developing reliable biochemical assays to measure lipid transport with high precision. In PNAS, Chua et al. (6) described a proteoliposome-based assay to monitor vectorial lipid flipping mediated by one of the most pharmacologically relevant MFS lipid transporters, the MFS domain containing 2a (Mfsd2a) lysolipid symporter.

Multiple reports have attributed MFS transporters an important role in the translocation of lipids across cell membranes, including, the lysophospholipid transporter (LplT) involved in lipids recycling in gram-negative bacteria (7); the mammalian sphingosine-1-phosphate transporters Spns2 (8) and Mfsd2b (9); the lysosomal lysophosphatidylcholine (LPC) and lysophosphatidylethanolamine transporter Spns1 (10); the gentiobiosyl-diacylglycerol transporter LtaA, involved in cell wall synthesis in *Staphylococcus aureus* (2); and the mammalian transporter Mfsd2a, a sodium-dependent LPC transporter, essential for brain and eye uptake of the omega-3 fatty acid docosahexaenoic acid (DHA) (3, 4, 11, 12).

In endothelial cells of the blood–retina and blood–brain barriers (BBB), Mfsd2a facilitates sodium-dependent uptake of DHA in the form of LPC (12, 13), a highly relevant biological process due to the absence of the protein machinery required for the synthesis of DHA de novo in brain and eye. DHA accounts for about one-fifth of the total membrane fatty acids in the central nervous system, and it is known to be essential for function and development. Both of these organs demand high uptake of systemic and dietary sources of DHA (13). Human loss-of-function mutations in Mfsd2a have been shown to cause autosomal recessive primary microcephaly, a disease characterized by hypomyelination, intellectual and developmental delay, and severe microcephaly. On the other hand, the downregulation of Mfsd2a expression levels is associated with an age-related increase in BBB permeability to neurotoxic proteins from plasma, which might be related to the progression of neurodegenerative diseases (13). In placenta, Mfsd2a is involved in the transport of DHA from the maternal circulation to the fetus. When this transport process is impaired, it can lead to gestational diabetes mellitus (13). Furthermore, beyond its role as a transporter, Mfsd2a acts as a receptor of endogenous retroviral proteins that mediate cell–cell fusion essential for the formation of the multinucleated-epithelial layer that constitutes the maternal–fetal interface. In this fusion process, syncytin (SYNC2), an envelope glycoprotein of retroviral origin encoded by a human endogenous retrovirus that entered the simian lineage more than 40 million years ago, binds selectively to Mfsd2a undergoing large conformational changes that result in fusion of cellular membranes (14).

In PNAS, Chua et al. described a proteoliposomes-based assay to monitor directional lipid flipping mediated by one of the most pharmacologically relevant MFS lipid transporters, the MFS domain containing 2a (Mfsd2a) lysolipid symporter.

The significance of Mfsd2a in increasing the BBB permeability to DHA is contrasted by its role as an inhibitor of transcytosis (12, 13). Studies involving Mfsd2a knockout mice have shown increased rates of transcytosis across the endothelial cytoplasm, without apparent tight junction disruption (11). The role of Mfsd2a in actively preventing transcytosis arises from inhibiting the formation of caveolae, presumably by affecting the lipid composition of the bilayer (11, 12–14). Therapeutic-drug delivery across the BBB could contribute to the treatment of neurological and neurodegenerative diseases affecting the central nervous system (12, 13). Thus, inhibitors targeting Mfsd2a are promising pharmacologically relevant molecules that could contribute to manipulating the permeability of the BBB to facilitate drug delivery. However, selective Mfsd2a inhibitors that could play this part have not been reported. Reliable in vitro lipid transport assays for high-throughput screening of this type of molecules would advance the discovery of drugs targeting Mfsd2a.

**Fig. 1.**
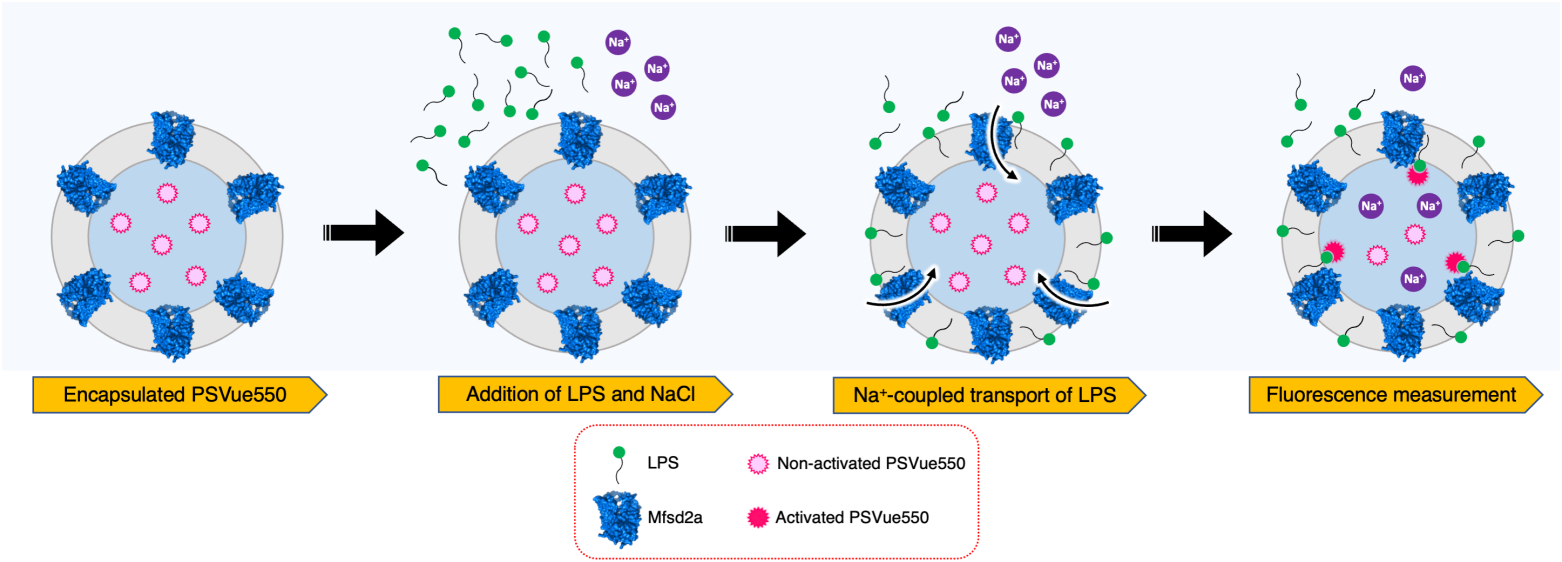
Schematic description of the LPS flipping assay. Mfsd2a proteoliposomes encapsulating PSVue550 are incubated in the presence of LPS and NaCl. Sodium-coupled LPS flipping catalyzed by Mfsd2a, expose the phosphoserine headgroup of LPS at the inner leaflet of the bilayer. Binding of PSVue550 to LPS headgroups results in fluorescence emission at 610 nm.

Previously, cell- or spheroplast-based assays using fluorophore-conjugated lipids have been very useful in detecting lipid transport activity of MFS transporters (7, 12). However, proteoliposome-based assays that measure lipid translocation without the interference of other proteins in the surrounding membrane have only been established for the gentiobiosyl-diacylglycerol transporter LtaA (2, 15), although relying on fluorophore-conjugated lipids. In PNAS, Chua et al. (6) established a proteoliposome-based assay where labeling of the lipid occurs in a posttranslocation reaction. This assay represents a significant advance in the study of Mfsd2a since it avoids the use of fluorophore-conjugated lipids, and it has the potential to be implemented in high-throughput screenings of inhibitory molecules. The assay is based on the encapsulation of PSVue550, a zinc(II)-dipicolylamine molecular probe that binds specifically to the phosphoserine headgroup of phosphatidylserine (PS) (Fig. 1) (16). Studies by Rice et al. (16) have shown that upon binding to PS, PSVue550 increases its emission intensity at 610 nm, when excited at 550 nm. Chua et al. (6) take advantage of the low membrane permeability of PSVue550 to trap this molecule inside proteoliposomes carrying Mfsd2a (Fig. 1). Since Mfsd2a present relaxed selectivity toward a variety of lysolipids carrying esterified zwitterionic headgroups (e.g., lysophosphatidylserine, LPS), Chua et al. apply LPS to the external buffer of the proteoliposomes detecting an increase in PSVue550 fluorescence (6). In contrast, protein-free liposomes, proteoliposomes of the melibiose transporter MelB, and proteoliposomes of a Mfsd2a variant carrying a mutation that disrupts sodium binding (D92A) display only background emission levels (4, 6). Thus, the assay developed by Chua et al. 1) provides direct evidence that Mfsd2a exhibits flippase activity leading to vectorial translocation of LPS toward the inner leaflet of the proteoliposomes membrane, 2) confirms that LPS transport occurs in a sodium-dependent manner, and 3) shows that LPS moves along the sodium concentration gradient. In addition, Chua et al. use their in vitro assay to estimate apparent affinity constants (Km) for LPS, and corroborate that an acyl chain of 14 carbons is required for LPS flipping (6).

The assay developed by Chua et al. has great potential to be used for the selection of molecules inhibiting Mfsd2a flippase activity. In this regard, it would be extremely interesting to show whether SYNC2 inhibits Mfsd2a transport in the experimental set-up established by Chua et al., and how this compares with previous reports where cell-based assays have shown that SYNC2 abolishes LPC transport with a half-maximal inhibitory concentration in the nanomolar range (5). Further studies aiming to optimize the inhibitory properties of this type of molecules are highly desirable.

Recent high-resolution structures of Mfsd2a (3–5) and LtaA (2, 15), revealed that although the lipid substrates of these two MFS transporters are different, both proteins share architectural similarities, the most important of which is an amphipathic central cavity that is predicted to be also present in the bacterial LplT (7) and in other MFS lipid transporters. The amphipathic nature of the central cavity suggests a common mechanism of substrate recognition and translocation, which Lambert et al. describe as a “trap-and-flip” mechanism, in which the lipid substrate would be enclosed entirely into the central cavity of the transporter, before being delivered to the opposite membrane leaflet (15). However, how does lipid recognition occur? How does the lipid flips in the central cavity? What stages of the translocation cycle are the most relevant? Answering these questions will help us to reveal the mechanism of lipids transport by Mfsd2a, provide a foundation for the design of delivery strategies for amphipathic drugs across the BBB, and will contribute to discovering molecules with inhibitory characteristics. The study by Chua et al. (6) tackles some of the questions mentioned above by performing mutagenesis analysis and cell-based transport assays. They reveal that polar residues close to the extracellular side of Mfsd2a are important for sodium and LPC recognition, and that hydrophobic residues located in the hydrophobic part of the central cavity participate in the capture of LPC, which may constitute an important step in the reorientation (flip) of the LPC molecule from a headgroup-up to a headgroup-down conformation. The results obtained by Chua et al. agree with a trap-and-flip model of LPC transport, in which the transition between inward- and outward-facing states might follow a “rock-and-swing” mechanism, as proposed by Martinez–Molledo et al. (5).

Together, the findings reported by Chua et al. provide technical guidance to assess LPS transport activity catalyzed by Mfsd2a. In addition, this assay poses the potential to be further developed into a high-throughput screening technique for the selection of inhibitory molecules targeting Mfsd2a. Their results reaffirm that Mfsd2a catalyzes vectorial transport of LPS coupled to a sodium concentration gradient, and deepen our understanding of the mechanism of a major pharmacologically relevant MFS transporter. Furthermore, the assay developed by Chua et al. could be adapted to study the lipid transport activity of other MFS lipid transporters such as, Spns1, Spns2, and Mfsd2b.
